# Supplementary material for: Quality of adolescent and youth-friendly health services in Dehana district public health facilities, northeast Ethiopia: Using the Donabedian quality framework
Source: PLoS One. 2021 Oct 20;16(10):e0258718. doi: 10.1371/journal.pone.0258718 (PMC8528293; doi:10.1371/journal.pone.0258718)
Supplement: Attachment — Submitted filename: Point-by-point responses (PONE-D-20-35107).docx [file pone.0258718.s002.docx]

Date: August 24, 2021

**To:** Editor in-chief of PLOS ONE Journal

**Subject:** Submission of a revised manuscript for publication

**Title:** “Quality of adolescent and youth-friendly health services in Dehana district public health facilities, northeast Ethiopia: Using the Donabedian quality framework." [Manuscript ID: PONE-D-20-35107]

Dear Editor,

Thank you for giving me the opportunity to submit a revised draft of my manuscript titled [mention the manuscript’s title] to [include the name of the journal, italicized].

Firstly, we would like to appreciate and thank the academic editor and reviewers for investing their time and energy to review and make comments. It is with great pleasure to receive the invaluable and constructive comments which improves our manuscript.

We accepted and tried to incorporate all of the comments provided. Thus, the comments are attached here below with their point-by-point responses. In addition, the detailed changes made are highlighted in the “revised manuscript with track changes” and also the clean copy of the revised manuscript is prepared.

Finally, we kindly request you to review our point-by-point responses to the editors’ and the reviewers’ comments and concerns below.

**Comments from Editor:**

Journal requirements: When submitting your revision, we need you to address these additional requirements.

**Comment 1:** Please ensure that your manuscript meets PLOS ONE's style requirements, including those for file naming. The PLOS ONE style templates can be found at <https://journals.plos.org/plosone/s/file?id=wjVg/PLOSOne_formatting_sample_main_body.pdf> and https://journals.plos.org/plosone/s/file?id=ba62/PLOSOne_formatting_sample_title_authors_affiliations.pdf

**Authors response:** Thank you so much for your comments. The comment is accepted and corrected in the style of the PLOS ONE journal. Please refer to the clean version of the revised manuscript.

**Comment 2:** Please include additional information regarding the quantitative survey or qualitative questionnaire used in the study and ensure that you have provided sufficient details that others could replicate the analyses. For instance, if you developed a questionnaire as part of this study and it is not under a copyright more restrictive than CC-BY, please include a copy, in both the original language and English, as Supporting Information, or include a citation if it has been published previously."

**Authors response:** Dear the editor, thank you for your concerns. the quantitative survey questionnaires for this study were adopted from the WHO and the federal ministry of health of Ethiopia and we citated for this. The semi-structured guiding questions were designed by the principal investigator by considering existing literatures and then five experts (program experts and researchers) who have more than 3 years work experience in the aera were reviewed and necessary modifications were made. In addition, a pre-test was done by the same data collectors who collect the final data and a final semi-structure interview guide was established to be used in the study as an instrument.

**Comment 3:** "In the Methods, please clearly discuss how the questionnaire was validated and pre-tested."

**Authors response:** Thank you for this suggestion. We tried to address your concerns. Please refer the clean version of the revised manuscript on page 9-10, lines 167-190.

**Comment 4:** “In your Methods section, please provide additional information about the participant recruitment method and the demographic details of your participants. Please ensure you have provided sufficient details to replicate the analyses such as: a) a description of any inclusion/exclusion criteria that were applied to participant recruitment, b) a statement as to whether your sample can be considered representative of a larger population, c) a description of how participants were recruited, and d) descriptions of where participants were recruited and where the research took place.

**Authors response:** We all authors would like to thank you for your insights and comments. We include all the points raised. We kindly invite you to see the revised manuscript on the methods section of the revised clean version manuscript.

**Comment 5:** We note that the grant information you provided in the ‘Funding Information’ and ‘Financial Disclosure’ sections do not match. When you resubmit, please ensure that you provide the correct grant numbers for the awards you received for your study in the ‘Funding Information’ section.

**Authors response:** Thank you very much and we appreciate your insights. We tried to address the issues.

**Comment 6:** We note that you have indicated that data from this study are available upon request. PLOS only allows data to be available upon request if there are legal or ethical restrictions on sharing data publicly. For more information on unacceptable data access restrictions, please see <http://journals.plos.org/plosone/s/data-availability#loc-unacceptable-data-access-restrictions>.

**Authors response:** Thank you for your comments, we are tried to address the issue as to the PLOS ONE data sharing policy.

**Comment 7:** In your revised cover letter, please address the following prompts:

a) If there are ethical or legal restrictions on sharing a de-identified data set, please explain them in detail (e.g., data contain potentially sensitive information, data are owned by a third-party organization, etc.) and who has imposed them (e.g., an ethics committee). Please also provide contact information for a data access committee, ethics committee, or other institutional body to which data requests may be sent.

b) If there are no restrictions, please upload the minimal anonymized data set necessary to replicate your study findings as either Supporting Information files or to a stable, public repository and provide us with the relevant URLs, DOIs, or accession numbers. For a list of acceptable repositories, please see http://journals.plos.org/plosone/s/data-availability#loc-recommended-repositories.

We will update your Data Availability statement on your behalf to reflect the information you provide.

**Authors response:** Dear editor thank you for your comments and concerns. All data generated or analyzed during this study are included in this manuscript.

**Comment 8:** Please review your reference list to ensure that it is complete and correct. If you have cited papers that have been retracted, please include the rationale for doing so in the manuscript text, or remove these references and replace them with relevant current references. Any changes to the reference list should be mentioned in the rebuttal letter that accompanies your revised manuscript. If you need to cite a retracted article, indicate the article’s retracted status in the References list and also include a citation and full reference for the retraction notice.

**Authors response:** Dear editor thank you for your critical insights. We reviewed our reference lists and tried to correct all the retracted articles.

**Comments from Reviewer 1**

**Comment:** Accept with no comments

**Authors response:** Thank you.

**Comments from Reviewer 2**

**Comment 1:** Firstly, thank you for inviting me to review this article, it made for interesting reading. While the study is significant and the findings are interesting, I am unsure about the novelty of the manuscript and whether it adds more to the existing literature on the same topic of interest. That being said, it is a well written article and I applaud the authors for their efforts to increase awareness regarding a significant section of the population, the health care services available for adolescents and their inadequacies. If similar studies using the same quality assessment tools have been conducted before, what is the justification for conducting this study? Is there a novel component to this study that merits publication and dissipation of findings to the scientific community?

**Authors response:** Dear reviewer thank you for your comments and suggestions. We tried to address all your concerns. In Ethiopia, there have been limited studies on quality of AYFS, and those studies done so far were primarily focused on identifying factors associated with youth friendly services utilization. Moreover, youth friendly service quality related studies so far were not supported with qualitative methods. Whereas, the current study applies a mixed study design and the Donabedian three-dimension quality framework to assess the quality of AYFS, that will provide a more detailed information than previous studies.

**Comment 2:** The English could be improved in the introduction part of the article, there are several places where authors have missed out grammatical errors.

**Authors response:** Dear reviewer, thank you so much for your comments and suggestions. The manuscript has been revised by English language expert and grammar and spellings have been improved throughout the manuscript, and we made rewording and rephrasing some parts of the paragraphs of the paper accordingly; please see the clean version of the revised manuscript once again.

**Comment 3:** In the first paragraph of the introduction, the authors can mention common causes of mortality in adolescents and youth in addition to risky behaviors.

**Authors response:** Dear reviewer, thank you for your insights. We have addressed the issue; please see the clean version of the revised manuscript on page 7, lines 59-65.

**Comment 4:** It would be more appropriate to mention 'target population' than 'source population' in the methods part of the article. This is just a suggestion for the authors to consider.

**Authors response:** Thank you for this suggestion. We accept your comment and replace the source population with target population. Please see the revised clean version manuscript on page 7, line 116

**Comment 5:** Was the investigator who collected qualitative data trained in qualitative research beforehand? What was the exact method used? Please mention in-depth interviews if that was the method chosen to collect qualitative data.

**Authors response:** Dear reviewer, thank you for your comments. To collect the qualitative data, we used key informant interview not in-depth interviews. This is because of the nature of the study which needs a detailed and rich information on the availability of resources and health facility management issues related to AYFS. As result, five health center heads and four AYFHS provider coordinators were purposively selected as a key informant. please see the clean version of the revised manuscript on page 8, lines 135-141

**Comment 6:** Was the validity and reliability of the Amharic translated version of the semi-structured questionnaire checked? If it was, please report the values in terms of Cronbach's alpha and other appropriate measures. If it was not, what is the justification for not checking the validity and reliability?

**Authors response:** Dear reviewer, thank you for your concern and valuable comments. The overall internal reliability of the the quantitative survey tools was checked using Cronbach’s alpha reliability test with a score of greater than 0.81. The tool was pre-tested on 21 clients at the Asketema health center in Gazgibla district (the nearby districts) to make sure the study's internal validity and the guiding questions were designed principal investigator and experts (program experts and researchers) who have more than 3 years’ work experience in the aera were reviewed it. Besides, a pre-test was done for the interview guiding questions by the same data collectors who collect the final data. please refer to the clean version of the revised manuscript on page 10, lines 177-184

**Comment 7:** There are some grammatical errors in the discussion as well, authors can proof read and make corrections accordingly.

**Authors response:** Dear reviewer, thank you for your insights, we have done thorough English editing and corrected the grammatical mistakes in the revised manuscript. please see the clean version of the revised manuscript.
